# Surgical repair of massive dilatation of the right atrium with tricuspid regurgitation

**DOI:** 10.1186/s13019-018-0769-7

**Published:** 2018-07-03

**Authors:** Masaho Okada, Hirotaka Watanuki, Kayo Sugiyama, Yasuhiro Futamura, Katsuhiko Matsuyama

**Affiliations:** Department of Cardiac Surgery, Aichi Medical University, 1-1 Yazakokarimata, Nagakute, Aichi Japan

**Keywords:** Massive dilatation of right atrium, Atrial fibrillation, Right atrial plication, Tricuspid valve repair, Tricuspid annular plane systolic excursion

## Abstract

**Background:**

Massive dilatation of the right atrium with tricuspid regurgitation is frequently diagnosed by accidental recognition of an enlarged cardiac silhouette during routine chest radiography. Although some patients are asymptomatic, enlargement of the right atrium can cause secondary tricuspid regurgitation due to dilatation of the tricuspid annulus, associated with arrhythmias and thrombus formation leading to pulmonary embolism, stroke, and, rarely, sudden death due to left ventricular compression.

**Case presentation:**

A 76-year-old woman was followed up due to atrial fibrillation and tricuspid regurgitation for 8 years. A follow-up echocardiogram showed progressive dilatation of the right atrium. Because of the development of shortness of breath, right atrial plication and tricuspid valve repair were performed. Tricuspid annuloplasty was performed on the beating heart with the use of a 28-mm Carpentier-Edwards Physio tricuspid annuloplasty ring. Plication of the enlarged right atrium was performed at the interatrial septum, the free right atrium wall including the appendage, and the space between the inferior vena cava and the tricuspid ring. Closure of the left atrial appendage was performed from outside to prevent left atrial thrombus formation. Postoperative X-ray and computed tomography showed reduced cardiac silhouette and right atrial volume. The patient was discharged uneventfully and returned for follow-up visits with improved symptoms.

**Conclusions:**

An adult case of massive dilatation of the right atrium of unknown etiology is reported. The patient’s symptoms were relieved by our operative procedure.

## Background

Massive dilatation of the right atrium (RA) with tricuspid regurgitation (TR) is frequently diagnosed by accidental recognition of an enlarged cardiac silhouette during routine chest radiography. Although some patients are asymptomatic, TR is associated with arrhythmias and thrombus formation leading to pulmonary embolism, stroke, and, rarely, sudden death due to left ventricular (LV) compression [1]. TR may rarely cause massive dilatation of the RA. Here we report a surgical case of massive dilatation of the RA with severe TR.

## Case presentation

**Fig. 1:**
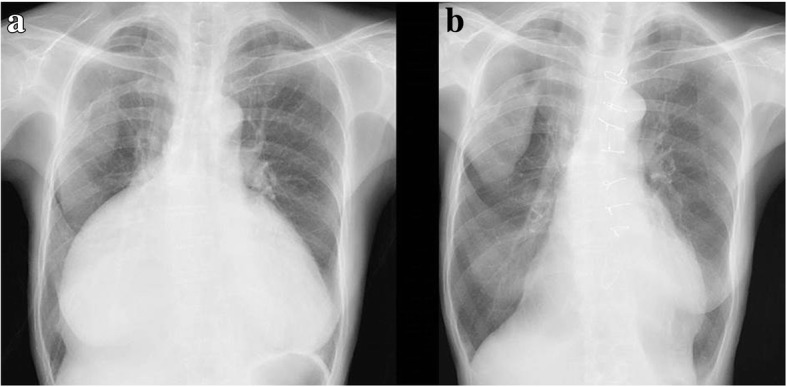
**a** Chest X-ray at preoperative examination showed severe enlargement of the heart, with a cardiothoracic ratio of 88%. **b** Postoperative chest X- ray showed reduced right-side shadow of the cardiac silhouette

**Fig. 2:**
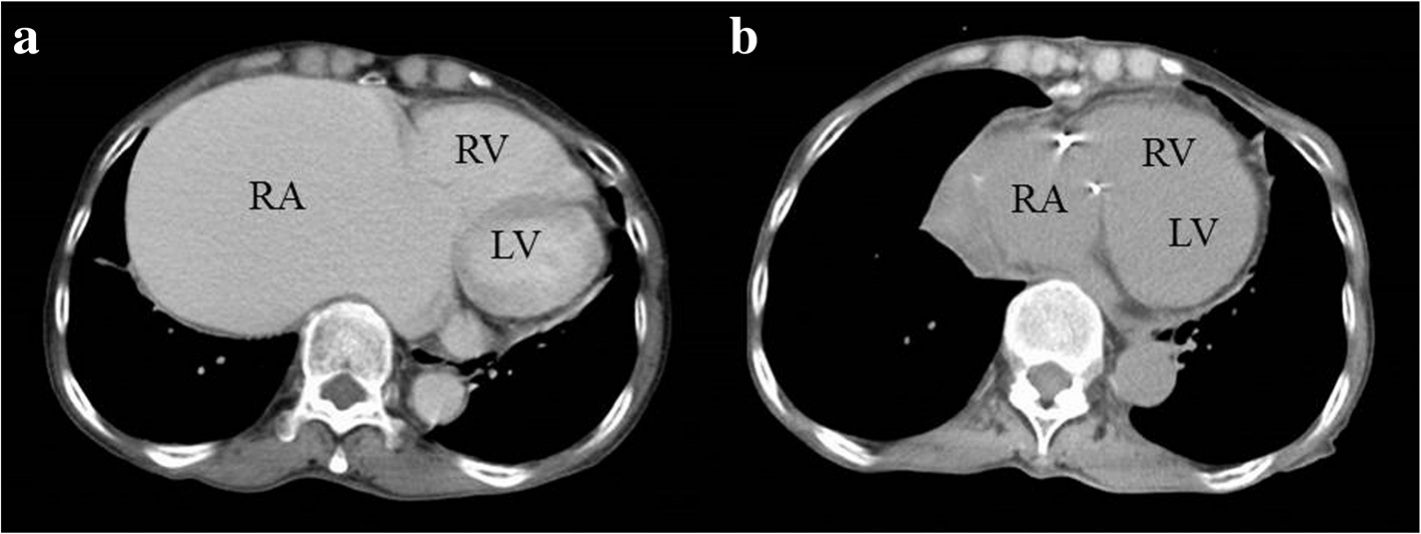
**a** Computed tomography (CT) findings showed that the size of the RA increased gradually up to 121 mm 1 year previously. **b** Postoperative CT scan showed reduction of the RA. LV, left ventricle; RA, right atrium; RV, right ventricle

**Fig. 3:**
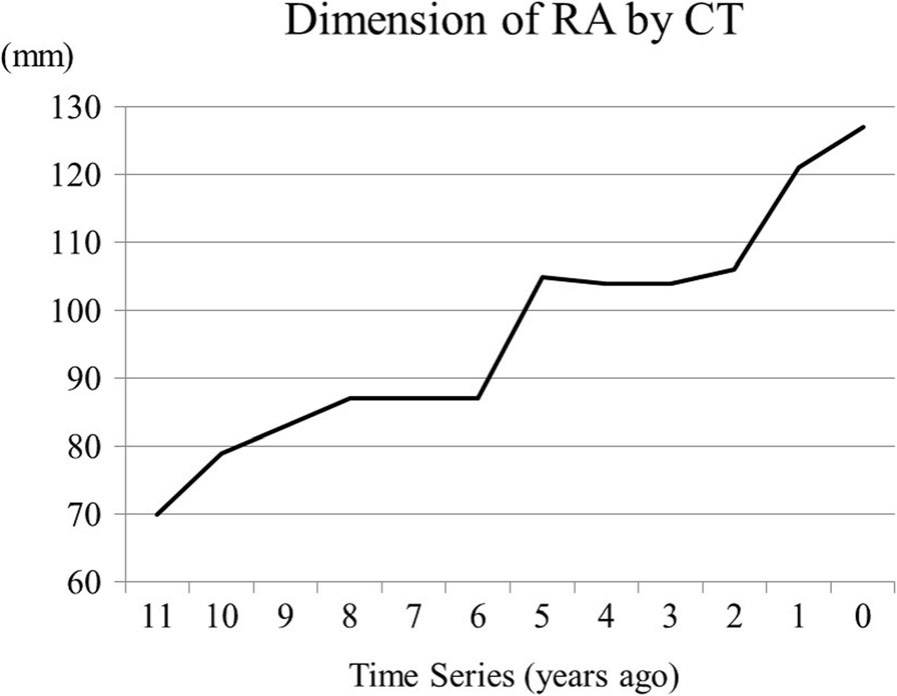
The change in dimension of the right atrium (RA) by computed tomography (CT) showed that the size of the RA increased with time

A 76-year-old woman had an 8-year history of atrial fibrillation (AF) and severe TR. Her history included mild hypothyroidism and right upper lobectomy for lung carcinoma 11 years previously. For 2 years, she had complained of shortness of breath when lying in the left lateral decubitus position. She had felt dyspnea after mild exercise for 9 months. Recently, she had a sense of abdominal fullness. Although administration of diuretics was started, her symptoms did not completely improve, and she was referred to our department for surgical treatment. The follow-up chest X-ray showed a gradually protruding right-side shadow of the cardiac silhouette, and the cardiothoracic ratio on the chest X-ray reached 88% (Fig. 1a). The electrocardiogram showed AF with low fibrillatory wave amplitude. Echocardiography showed an enlarged right ventricular (RV) cavity and mild paradoxical motion of the ventricular septum. The tricuspid valve had no findings of an organic and constructive abnormality, with no severe tethering. The annular size of the tricuspid valve was 50 mm, and the tricuspid annular plane systolic excursion was 21 mm (Table 1). Repeated preoperative cardiac catheterization showed slightly elevated wedge pressure with mild pulmonary hypertension, although LV function was preserved with a cardiac index of 4.0 (Table 2). There was no L-R shunt disease. Computed tomography (CT) findings showed that the maximum size of the RA reached 121 mm (Fig. 2a). The change in dimension of the RA by CT showed that the size of the RA increased with time (Fig. 3). Blood tests showed no liver dysfunction.

**Table 1:** Preoperative and postoperative echocardiography findings

Measurement	Years before presentation							
	6	4	3	2	1.5	0.5	Pre-ope	Post-ope
LVDd (mm)	37.1	39.5	36.6	40.4	38.8	43.2	37.6	42.4
LVDs (mm)	25.4	29.3	25.2	23.1	28.3	29.9	21.9	29.7
LVEF (%)	60.5	51.4	59.7	74.5	53.5	58.7	73.5	57.5
LAD (mm)	36	34.9	40.3	44.8	33.9	40.4	38.9	43.3
RA (mm)	81	91	86	94.6	120	96.6	109	33.5
TAPSE (mm)	19.9	19.2	23.7	22	14.8	17.7	21.1	11.2
IVC (mm)	21.2	–	19.8	26.5	20.2	24.8	26	16.1
TR grade	Severe	Severe	Severe	Severe	Severe	Severe	Severe	Mild

*Pre-ope* preoperative, *Post-ope* postoperative, *LVDd* left ventricular end-diastolic dimension, *LVDs* left ventricular end-systolic dimension, *LVEF* left ventricular ejection fraction, *LAD* left atrial dimension, *RA* right atrium, *TAPSE* tricuspid annular plane systolic excursion, *IVC* inferior vena cava, *TR* tricuspid regurgitation

**Table 2:** Cardiac catheterization analysis

Measurement	Years before presentation	
	4 years	Pre-ope
RA mean pressure (mmHg)	5	9
RV systolic pressure (mmHg)	16	27
RV diastolic pressure (mmHg)	3	3
PA systolic pressure (mmHg)	21	31
PA diastolic pressure (mmHg)	12	17
PCWP (mmHg)	6	13
Systemic mean artery pressure (mmHg)	83	87
Cardiac index (l min^−1^ m^−2^)	4.42	4.02

*RA* right atrium, *RV* right ventricle, *PA* pulmonary artery, *PCWP* pulmonary capillary wedge pressure

**Fig. 4:**
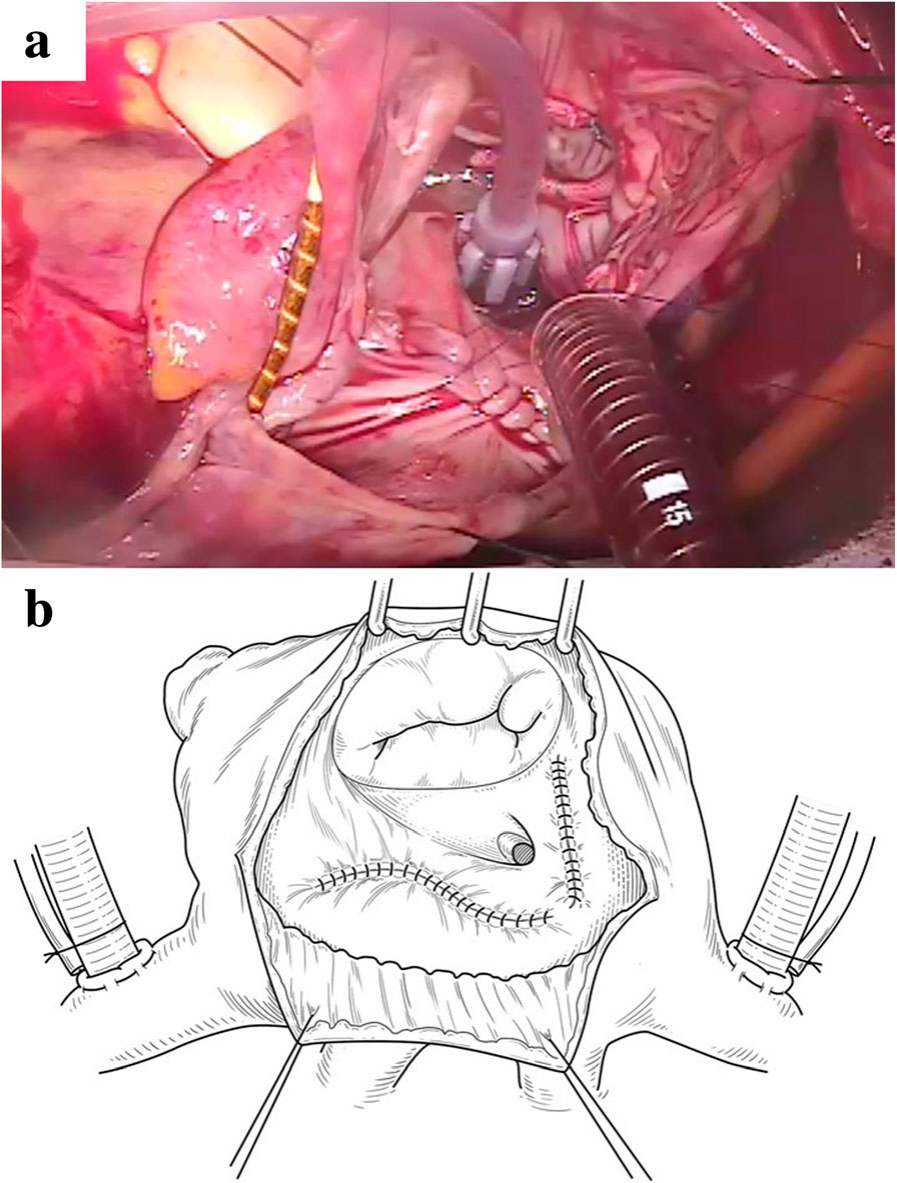
**a** Intraoperative findings. Right atrium (RA) plication was performed at the interatrial septum and the space between the inferior vena cava and the tricuspid ring. **b** Intraoperative findings for RA inner side plication

**Fig. 5:**
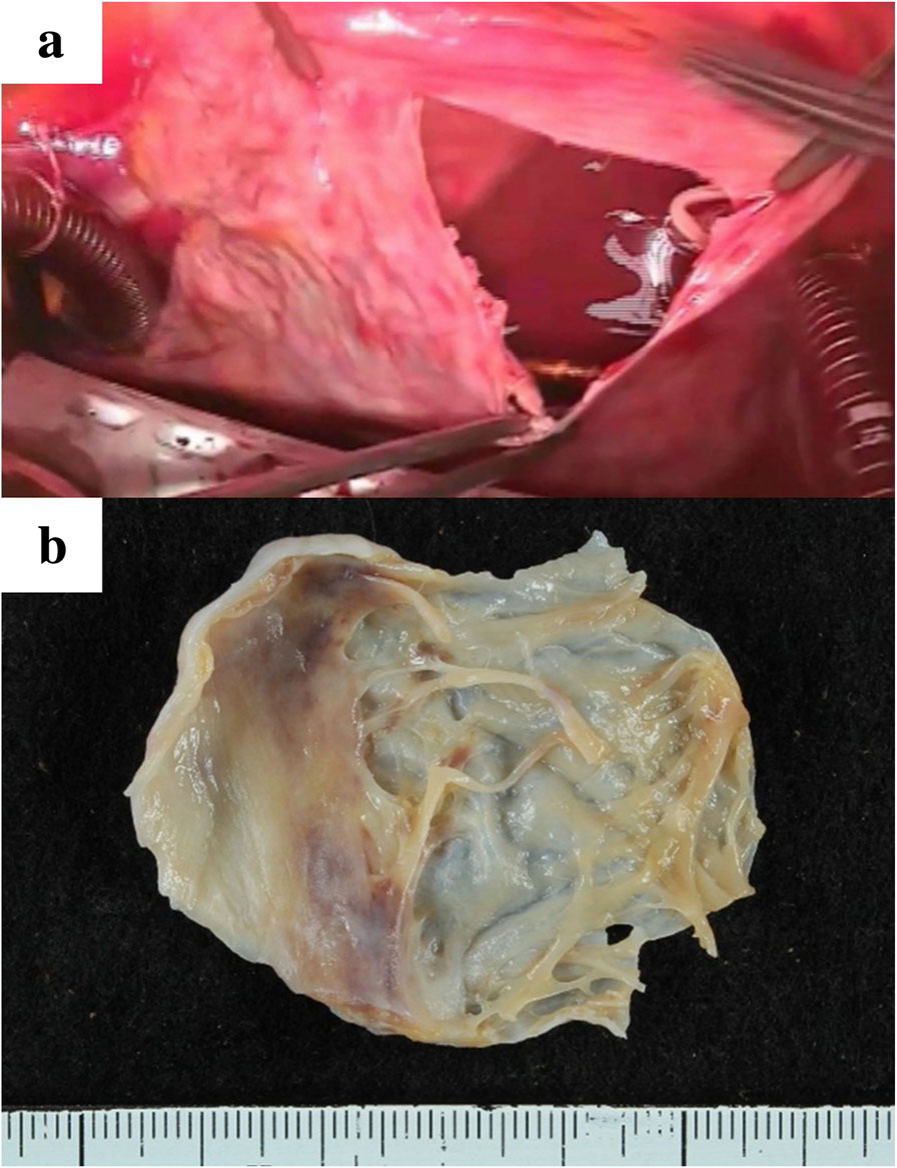
**a** Photograph taken just after cutting of the right atrium (RA) wall during operation shows the extremely thin RA wall. **b** Tissue specimen of the resected free wall of the RA

**Fig. 6:**
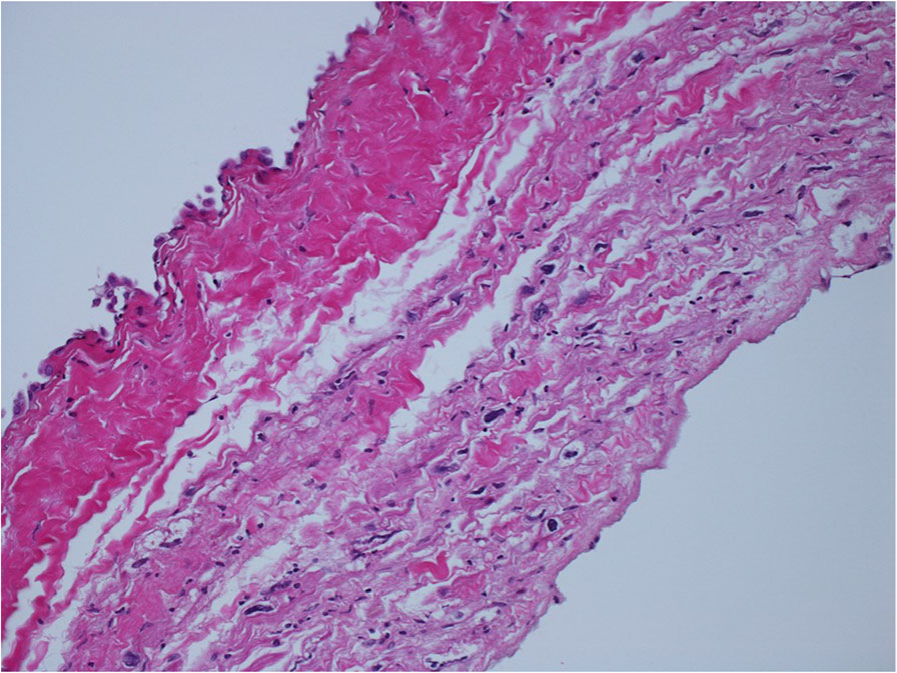
Microscopic examination of tissue specimen from the right atrium (RA) wall showed thinning of the myocardium, inflammatory cell infiltrate, and few cardiomyocytes

Surgery was performed via median sternotomy. The pericardium was extremely thin on the RA side without any defect. There was no adherence in the pericardial cavity. Cardiopulmonary bypass was established by ascending aorta cannulation with bicaval drainage. The RA was extremely thin and the tricuspid valve annulus was enlarged, with a diameter of 55 mm, but there was no tricuspid structural abnormality. Tricuspid annuloplasty was performed on the beating heart using a 28-mm Carpentier-Edwards Physio tricuspid annuloplasty ring (Edwards Lifesciences, Irvine, CA, USA). Plication of the enlarged RA was performed, mainly at the interatrial septum, the free RA wall, including the appendage, and the space between the inferior vena cava and the tricuspid ring, in addition to the free RA wall, including the appendage (Fig. 4a, b). Additionally, closure of the left atrial appendage from the outside was performed to prevent left atrial thrombus formation. All procedures were performed on the beating heart. The postoperative course was uneventful. The pathological findings of the RA wall demonstrated thinning of the myocardium, inflammatory cell infiltrate, and few cardiomyocytes (Figs. 5a-b, 6). Postoperative X-ray and CT showed reduced cardiac silhouette and RA volume (Figs. 1b, 2b). Postoperative echocardiography showed an ejection fraction of 58% and mild TR with a pressure gradient of 29 mmHg (Table 1). The postoperative value of tricuspid annular plane systolic excursion (TAPSE) decreased after the operation. However, the patient’s symptoms were completely resolved, and she was discharged 3 weeks after surgery. The patient is doing well 2 years after surgery.

## Discussion

The pathophysiology of dilated RA is occasionally combined with long-standing AF and severe TR. The etiology of the massive RA dilatation in our patient was unknown. The RA pressure in our patient was less than 10 mmHg. The pathological findings include thinning RA wall and few cardiomyocytes, and suggest that the etiology was the original weakness and the acquired degenerative process of the RA. Reported symptoms of enlarged RA include dyspnea, palpitations, chest pain, right heart failure, syncope, and fatigue. However, half of patients with enlarged RA are asymptomatic at the time of diagnosis [1–3].

The approach to treatment of isolated severe TR with dilatation of the RA is controversial. Surgical treatment is recommended in the presence of arrhythmias, congestive heart failure, or LV compression, which may cause sudden death [1]. In the majority of cases, asymptomatic patients can be treated conservatively. The operative indications for TR are mainly related to the presence of symptoms and RV function. According to the guidelines, tricuspid valve (TV) surgery is indicated for symptomatic patients with primary TR unless there is severe RV dysfunction [4]. In our patient, surgery was indicated due to progressive dilatation of the RA, a sign of LV compression, the presence of symptoms, and less severe RV dysfunction.

On the other hand, TV surgery for asymptomatic patients with progressive RV dysfunction is controversial. RV function is a critical component of overall cardiac function, with prognostic value for predicting symptomatic limitations and outcome, although it is difficult to assess RV function because of the complex geometry of the right ventricle. TAPSE has been proposed as a simple and reproducible parameter for the evaluation of RV function and is recommended in the recent guidelines for echocardiographic quantification of RV function, which show that values less than 16 mm indicate RV systolic dysfunction [5]. TAPSE can predict the postoperative outcome of TV surgery [6]. In our case, preoperative TAPSE remained within the normal range. However, postoperative TAPSE significantly decreased to 11.2 mm despite symptomatic improvement. If preoperative TAPSE is less than the normal range, TV surgery could decrease TAPSE further, resulting in a poor prognosis. TV surgery should be carried out early to avoid irreversible RV dysfunction with consideration for TAPSE.

Reduction atrioplasty is supposed to improve respiratory function by reducing RA volume and increasing tidal volume. Although the specific surgical technique for reduction atrioplasty was not described, Kalangos et al. reported reinforcement after partial resection by approximating and fixing the neighboring autologous pericardium to prevent the recurrence of dilatation [7]. In our patient, RA wall reinforcement with materials was not done, because the strength of the RA wall was sufficient after reduction atrioplasty.

Although left atrial was not enlarged, Maze procedure was not performed because of long-standing AF, very low fibrillatory wave amplitude, and irreversible RA function.

## Conclusions

A rare case of massive dilatation of the RA of unknown etiology is reported. Our operative procedure relieved the patient’s symptoms and helped to prevent future complications, such as arrhythmia and left heart failure due to dilatation of the right ventricle.

## Abbreviations

AF: Atrial fibrillation
CT: Computed tomography
LV: Left ventricular
RA: Right atrium
RV: Right ventricular
TAPSE: Tricuspid annular plane systolic excursion
TR: Tricuspid regurgitation
TV: Tricuspid valve
